# Spatial microbiome and synthetic community analyses reveal *Trinickia sclerotiorum* sp. nov. promotes sclerotia mortality of *Sclerotinia sclerotiorum*

**DOI:** 10.1093/ismejo/wrag201

**Published:** 2026-07-29

**Authors:** Zong-Han Shie, Hao-Xun Chang

**Affiliations:** Department of Plant Pathology and Microbiology. National Taiwan University. Taipei 106319, Taiwan; Department of Plant Pathology and Microbiology. National Taiwan University. Taipei 106319, Taiwan; Master Program for Plant Medicine, National Taiwan University, Taipei 106319, Taiwan; Center for Biotechnology, National Taiwan University, Taipei 106319, Taiwan

**Keywords:** 10× Genomics Xenium, bacteria–fungi interactions, plant pathogen, spatial microbiome, synthetic community (SynCom)

## Abstract

Bulk microbiome analysis obscures the spatial heterogeneity of microbial communities, limiting the understanding of bacterial distribution inside fungal sclerotia, which are the survival structures of the plant pathogen *Sclerotinia sclerotiorum*. Although studies have profiled microbiomes of different fungal structures, sclerotia microbiome remains largely unexplored. We applied bulk microbiome using PacBio full-length 16S rRNA sequencing, synthetic community (SynCom), and 10× Genomics Xenium platform to resolve the spatiotemporal contribution of soil bacteria to sclerotia mortality. In the bulk microbiome, the relative abundance of soil bacteria such as *Massilia* and *Trinickia* were enriched when sclerotia mortality increased under flooding conditions. Twelve enriched sclerotia-associated bacteria were assembled into a SynCom to study their causality for sclerotia mortality. Spatial microbiome analysis revealed the temporal dynamics and distribution pattern of each SynCom member. Among them, *Trinickia sclerotiorum* sp. nov. type strain MC (TsMC) was found to reach the most interior sclerotia and its relative abundance coincided with sclerotia mortality. TsMC exhibited the highest importance in four antagonistic assays and SynCom drop-out test. Whole genome sequencing confirmed TsMC as a novel species with antifungal potential. Collectively, we document a novel bacterium for sclerotia mortality and demonstrate the advantage of spatiotemporal distribution in microbiome research.

## Introduction

Bulk microbiome research in recent years has articulated a sustainable future of agriculture from diverse aspects, including plant disease management [1, 2], and many studies have shown the capacity of bulk microbiome in discovering antagonistic bacteria to counteract plant pathogenic fungi [3]. Although these studies have demonstrated the success of bulk microbiome, few have elucidated the dynamic movement of a microbial community in the antagonistic bacteria–fungi interaction. Similar research constraints used to be found in bulk transcriptomics, proteomics, or metabolomics, for which the experimental procedures average out signals from different cell types spatially distributed in a tissue, limiting the resolution of understanding gene, protein, or metabolite functions [4]. With the advent of single-cell and spatial technologies on multi-omics, many studies have advanced the mechanistic insights into not only medical diseases [5] but also plant diseases [6, 7]. However, one of the multi-omics lagging behind in this wave of technology breakthrough is microbiome, in which bacteria are the major targets of interest. One pioneer study embraced the power of the sequencing-based Visium platform by customizing a 16S/18S/ITS/poly-d(T) multimodal array to simultaneously detect both bacterial and fungal microbiome, together with *Arabidopsis* gene expression [8]. This study provides a proof-of-concept in the spatial correlation of microbes and plant defense-related gene *PR1*, but the discontinuity of this multimodal array by the manufacturer limits the broad application to explore spatial microbiome. A fortunate alternative strategy is the hybridization-based platform such as 10× Genomics Xenium and Vizgen MERSCOPE [9, 10], which allow *in situ* detection for the RNA of interest. Yet, the application has not been validated for spatial microbiome.

Instead of the spatial distribution of microbes, the strategic breakthrough on bulk microbiome in the past years was focused on the utilization of synthetic community (SynCom) to resolve the causality of microbes that cause symptoms or diseases [11, 12]. One of the classic SynCom studies uncovered the mechanism of *Arabidopsis* immune-related gene *rbohD* in handling dysbiosis and the opportunistic pathogen *Xanthomonas* strains [13]. Another classic study demonstrated the dysbiotic SynCom derived from *Arabidopsis mfec* mutant can lead to foliar chlorosis due to hyperdominant *Pseudomonadota* [14]. In contrast to the success of plant-associated SynCom, there are few examples of fungi-associated SynCom to tackle the causality in research. Another breakthrough on bulk microbiome was the adaptation of full-length 16S rRNA sequencing to replace the sequencing of short variable regions, for the better taxonomic resolution at species level [15, 16]. However, few bulk microbiome research in fungi has adopted the advantages of SynCom or full-length 16S rRNA. Although many studies have reported the bacterial biodiversity on fungal tissues such as mycelia [17], mushrooms [18], sporocarps [19], and perithecia [3], the fungal microbiome remains fragmented considering the integrative methodologies being applied to human and plant microbiomes.

One of the agriculturally important and specialized fungal tissues is sclerotium (pl. sclerotia), which can tolerate environmental stresses and persist in infected plants or infested soil for a long period and become the primary inoculum of a new farming season. Eliminating fungal sclerotia, via host genetic resistance [20], fungicides [21], biocontrol agents [22, 23], or agronomic practices such as flooding or tillage [24, 25], has been considered as a vital part of integrative disease management. Although several studies have identified antagonistic bacteria that can reduce the overall fungal growth or propagation, few have evaluated the microbial effect on sclerotia mortality. Therefore, this study integrates the PacBio full-length 16S rRNA sequencing, coupled with the fungi-associated SynCom and the Xenium platform for spatial microbiome, to illuminate the spatiotemporal dynamics of antagonistic bacteria invading sclerotia. The results discover bacteria with antagonistic potentials and demonstrate the feasibility and power of spatial microbiome in uncovering the antagonistic bacteria–fungi interactions.

## Material & methods

### Soil and fungal materials

Soil samples were collected from a rice–soybean rotation field at Taoyuan (24.96°N, 121.04°E) in Taiwan. The soil was processed by passing through a four-mesh sieve to remove debris and unify particle sizes. A portion of the soil was autoclaved at 121°C for two times, each time for 1 h with a 1-day interval, to eliminate most microbes. Both autoclaved soil and nonautoclaved soil were stored at room temperature before the experimental setup. Sclerotia of *Sclerotinia sclerotiorum* isolate 1980 [26] were utilized as inoculum. The fungal isolate was routinely maintained on Potato Dextrose Agar (PDA) (STBIO MEDIA, New Taipei City, Taiwan) and incubated at 25°C for 2 weeks to promote sclerotia formation. Mature sclerotia were harvested and desiccated for 2 days under sterile conditions in a laminar flow hood.

### Mortality assay

All materials used in the 3-month mortality assay, including plastic containers, lids, nonwoven fabric tea bags, and tissues, were sterilized by autoclaving at 121°C for 15 min. For heat-sensitive items, sterilization was conducted by UV-C irradiation for 30 min. Each experimental unit consisted of a plastic container with a nonwoven fabric tea bag, filled with a 1.25 ml teaspoon of sclerotia (~50 sclerotia). These sclerotia in a tea bag were buried in: (i) nothing as control (CK); (ii) autoclaved soil (AS); (iii) autoclaved soil with flooding (ASF); (iv) nonautoclaved soil (S); or (v) nonautoclaved soil with flooding (SF), with 5 cm of soil in depth, and maintained at 25°C for up to three months before evaluating their mortality rates (Fig. S1A). The soil treatment received 150 g of nonautoclaved or autoclaved soil, and the flooding treatment was supplemented with 50 ml of ddH₂O to achieve maximum water capacity. There were eleven burial timepoints, including 1-, 3-, 5-, 7-, 14-, 21-, 28-, 45-, 60-, 75-, and 90-day. Instead of repeated sampling from the same plastic container, there were five independent biological replicates (i.e. plastic containers) prepared for each timepoint under each treatment. Collectively, a total of 275 experimental units (5 treatments × 11 timepoints × 5 biological replicates) were placed following the Latin square design in a growth chamber at 25°C. At each timepoint, the sclerotia were retrieved from the tea bag. To remove most soil and contaminants, the sclerotia were washed with 10 ml of ddH₂O by vortex mixing (15 s), sonication (15 s), and additional vortex mixing (15 s), before drying on sterile tissue papers. This cleaning procedure was repeated twice, and then 30 sclerotia were assessed for mortality rate whereas the remaining sclerotia were stored at −80°C for subsequent DNA extraction.

For the mortality assay, the 30 sclerotia were evenly distributed into three water agar (WA) plates supplemented with antibiotics (0.1% rifampicin, streptomycin, and ampicillin) to inhibit bacterial contaminants. These WA plates were incubated at 25°C for 5 days, and the mycelia germinated from sclerotia were collected for molecular identification using the *S. sclerotiorum*-specific primers SSFWD and SSREV [27] (Fig. S1B). The presence of the correct amplicon served as the evidence of survived sclerotia, and the mortality rate (%) was calculated as:


$$\mathrm{Mortality}\ \mathrm{rate}\ \left(\%\right)=\left(1- \frac{\mathrm{Survived}\ \mathrm{sclerotia}}{10\ \mathrm{sclerotia}}\ \right)\times 100\%$$

For each biological replicate (i.e. a plastic container), the mortality rate was averaged from three WA plates as technical replicates. Mortality rates across the five treatments (CK, AS, ASF, S, and SF) and 11 timepoints were statistically analyzed to evaluate the effects of autoclaving, flooding, and their interaction at each timepoint.

### Microbiome analysis

For each biological replicate, five to six sclerotia were ground using a sterilized stainless-steel mortar and pestle in liquid nitrogen. Total DNA of sclerotia was extracted using the Presto™ gDNA Bacteria Advanced Kit (Geneaid Biotech, New Taipei City, Taiwan). The DNA quality and quantity were initially assessed by gel electrophoresis and the Nanodrop spectrophotometer (Thermo Fisher Scientific, Waltham, MA, USA). Bacterial full-length 16S rRNA was amplified by the primers 27F and 1492R from the PacBio library preparation kit. After excluding 5 low-quality samples, 65 samples in the ASF and SF groups at seven timepoints (1-, 3-, 5-, 7-, 14-, 21-, and 28-day) were sequenced using the PacBio Sequel IIe platform (BioTools, New Taipei City, Taiwan).

Raw sequences were processed with PacBio SMRT Link to generate HiFi reads (≥  Q30), which were filtered, trimmed, dereplicated, and denoised by the “DADA2” package v1.32.0 [28] to generate ASV (Fig. S1C). Chimeric ASVs identified by DADA2 and sequences not matching bacterial 16S rRNA sequences in the SILVA 16S database v138.2 [29] were removed. The remaining ASVs were aligned to the NCBI 16S RefSeq database using the BLAST+ v2.15.0, with a threshold at an E-value of 10^−5^. The top hit was selected following the lowest E-value, the highest Bit-score, and the highest identity [30]. The resulting taxonomy IDs were annotated by the “taxize” package v0.10.0 [31]. The ASVs were aligned using MAFFT v7.525 [32], and a maximum likelihood phylogenetic tree was constructed using the IQ-TREE2 v2.2.2.7 with the best-fit model (GTR + F + I + G4) [33]. Branch support was assessed with 1000 ultrafast bootstrap replicates.

The ASV table, taxonomy table, and phylogenetic tree were integrated by the “phyloseq” package v1.52.0 [34]. Samples were excluded if sequence depths <2000 reads [35] or insufficient coverage according to rarefaction curves estimated by the “vegan” package v2.7-1. All samples were rarefied to 5000 reads. For α-diversity, the Shannon Index was calculated using the “phyloseq” package. For β-diversity, NMDS were performed using the Bray–Curtis dissimilarity and the effects of timepoints and treatments were estimated by PERMANOVA using the “vegan” package. To visualize the microbial composition, the ASVs with average abundance <0.5% in the detected samples and occupancy <10% within each treatment (i.e. fewer than four samples) were filtered out, and all samples were normalized to the median sequencing depth. The relative abundances were plotted in a bar plot at the genus level and in a heatmap at the species level using the “plot_bar” function of the “microbiome” package. For differential abundance analysis between the ASF and SF treatments at each timepoint, all samples were normalized and analyzed using the “ANCOM” packages v2.10.0 [36] and “DESeq2” v1.48.1 [37].

### SynCom assembly and mortality assay of SynCom-inoculated sclerotia

The SF 5-day sclerotia were retrieved, cleaned, and ground with 2 ml of ddH_2_O. The ground aliquots were serially diluted with ddH_2_O and spread on different media, including Nutrient agar (HiMedia, Mumbai, India), 10% Tryptic soy agar (TSA) (STBIO MEDIA), Reasoner’s 2A agar (R2A) (HiMedia), and modified *Pseudomonas cepacia* azelaic acid tryptamine medium [38]. After culturing at 25°C for 7 days, morphologically distinct colonies were isolated and the full-length 16S rRNA sequences were Sanger-sequenced (Genomics Inc., New Taipei City, Taiwan). After the BLASTn searching against the database constructed by the SF 5-day microbiome ASVs (Fig. S1D), bacteria with 16S rRNA sequence identities over 97.0% were considered present in the microbiome. Subsequently, the full-length 16S rRNA sequences were BLASTn against the NCBI 16S rRNA RefSeq database, and taxonomic assignments were based on the top hit with ≥98.7% identity [39]. Strains with <98.7% identity were further compared against the broader NCBI nt database to infer their putative taxonomy. Twelve strains representing those bacterial genera enriched in differential abundance analyses were selected to assemble a SynCom mimicking the 5-day SF microbiome. Species-level identities were resolved using maximum-likelihood phylogenetic trees built from concatenated 16S rRNA–*gyrB*–*recA* sequences (for *Caballeronia, Cupriavidus, Paraburkholderia, Ralstonia*, and *Trinickia* strains) or concatenated 16S rRNA–*gyrB*–*lepA* sequences (for *Massilia* strains).

Bacterial suspensions of each SynCom member were harvested at OD_600_ = 0.5 and adjusted to 10^6^ CFU/ml with ddH₂O. Eight drop-out designs were assembled following a 2^3^ factorial scheme based on the presence or absence of three groups: (i) *Trinickia sclerotiorum* sp. nov. type strain MC (TsMC), (ii) *Massilia* sp. strain R20 together with *Massilia* sp. strain TB221 (2-Massilia), and (iii) the remaining nine SynCom members (9-member). The full SynCom was composed of equal volume of 12 bacteria members (1 × 10^6^ CFU/ml). In the drop-out test, each bacterium contributed 1/12 × 10^6^ CFU/ml, and the excluded proportion was replaced with an equal volume of ddH₂O. Ten sclerotia were co-cultured in 5 ml of these eight drop-out designs at 25°C in a 10 ml culture tube without shaking. After 1-, 3-, 5-, 7-, and 14-day, the sclerotia were retrieved for measuring mortality rate. The presence of germinating hyphae after incubation on WA for 5 days serves as evidence of survived sclerotia. The initial full SynCom assay was independently repeated three times, each with three biological replicates, and the following drop-out tests were independently repeated two times, each with three biological replicates.

### Sample processing for Xenium *in situ* platform

The control and the SynCom-treated sclerotia were co-cultured and harvested at 1-, 3-, 5-, 7-, and 14-day following an incubation for 5 days on WA plates. Subsequently, sclerotia were embedded in the Tissue-Tek O.C.T. Compound (Sakura Finetek, Torrance, CA, USA) within an aluminum mold, frozen in isopentane precooled by liquid nitrogen, and then stored at −20°C. Sections were cut at a thickness of 6 μm using the CryoStar NX50 (Thermo Fisher Scientific) at −10°C, mounted onto the capture area of Xenium slides, and subsequently stored at −80°C. This study included 29 SynCom-treated samples (*n* = 4, 6, 6, 6, and 7 biological replicates for 1-, 3-, 5-, 7-, and 14-day, respectively) and 2 control samples (*n* = 2 for 7-day).

A customized panel for the Xenium *in situ* single-cell spatial imaging platform v1 (10× Genomics, Pleasanton, CA, USA) was designed using the 10× Genomics Custom Panel Designer. The panel comprises 28 specific bacterial padlock probes targeting the 16S rRNA, *gyrB, recA*, and *lepA* gene transcripts across 12 SynCom members. To ensure binding specificity, all probe sequences underwent rigorous computational screening according to the supervision of 10× Genomics manufacturer to optimize the sequence of detection codewords, reduce cross-hybridization and off-target among SynCom members and *S. sclerotiorum*. Subsequently, sections were incubated at 37°C for 1 min, fixed in paraformaldehyde at room temperature for 30 min, and permeabilized using 1% SDS for 2 min followed by 70% methanol on ice for 60 min. Target-specific padlock probes were hybridized overnight at 50°C, followed by ligation (37°C, 2 h) and rolling circle amplification (30°C, 2 h) to generate high-density DNA nanoballs, following the manufacturer’s protocols. To mitigate background fluorescence, slides were treated with an autofluorescence quenching reagent before nuclei were counterstained with DAPI.

For Xenium data acquisition, slides were processed on the Xenium Analyzer, where regions of interest (ROIs) were defined based on the DAPI scan to cover the entire sclerotia section. The analyzer then executed cyclical rounds of fluorescently labeled oligo hybridization to the amplified DNA probes, followed by imaging and probe removal. This cyclic process generated unique optical signatures specific to each gene barcode, which were subsequently converted into gene identities. Transcript decoding was performed by mapping optical signatures to the custom panel design file (.json) using Xenium Onboard Analysis v4.0. A Q-Score threshold of 20 was applied to filter low-quality transcripts from the raw data.

### Spatial microbiome analysis for Xenium platform

Post-Xenium slides were stained to visualize the bacteria distribution. Stored slides were washed in 1X PBS-T buffer, and the fluorescence quencher was removed using 10 mM NaHS. After methanol fixation (10 min), slides were treated with crystal violet (7 g/L) and iodine solution, then decolorized with an isopropanol–acetone (1:1) mixture to achieve high contrast between bacteria and the tissue background. Stained sections were mounted in glycerol and scanned using an EasyScan Pro 6 whole-slide scanner (Motic Digital Pathology, Hong Kong, China).

Xenium data and crystal violet images were co-visualized using Xenium Explorer v4.1.0. The ROI tool was employed to manually delineate the sclerotia tissue boundaries, and the corresponding coordinates were exported. These coordinates were mathematically divided into five concentric zones of equal area, ranging from the sclerotia core (layer 1) to the periphery (layer 5) by the R package “sf” v1.0-24 [40]. The coordinates of these stratified zones were imported back into Xenium Explorer to extract the signal density (transcripts/mm^2^) for each target bacteria. To correct off-target noise, bacterial gene signal densities were corrected by subtracting the maximum signal density observed across the two noninoculated control replicates. Lastly, bacterial genes with a signal density below a threshold of 2 transcripts/mm^2^ were excluded. The bacteria *gyrB* gene was selected as the representative due to its high spatial concordance with bacterial aggregates visualized by crystal violet staining. The relative abundance of *gyrB* was plotted to illustrate the spatial microbiome over different time points (1–14 dpi) in five layers.

### Antagonistic assays

The antagonistic abilities of the 12 SynCom members and a reference bacterium *Escherichia coli* were evaluated using four phenotypes: (i) mycelia inhibition, (ii) germination inhibition, (iii) sclerotia-derived mycelia inhibition, and (iv) mycophagy, according to the following formulas.

To evaluate (i), a 5 mm mycelial plug from the actively growing margin of *S. sclerotiorum* was placed at the center of a plate. Each bacterium was precultured for 2 days and streaked 25 mm away from the mycelial plug on two sides. Colony diameter was measured on 1/10 PDA at 72 h postinoculation (hpi) and 1/10 TSA at 65 hpi.


(1) \begin{eqnarray*} \mathrm{Mycelia}\ \mathrm{inhibition}\ \left(\%\right)\!=\!\left(1{-}\frac{\mathrm{Colony}\ \mathrm{diameter}\ \mathrm{of}\ \mathrm{treatment}}{\mathrm{Mean}\ \mathrm{diameter}\ \mathrm{of}\ \mathrm{control}}\ \right){\times} 100\% \end{eqnarray*}

To quantify (ii), (iii), and (iv), bacterial suspensions were harvested at OD_600_ = 0.5 and adjusted to 10^8^ colony-forming units (CFU) per milliliter. A final concentration of 10^6^ CFU/ml in a 10 ml culture tube was co-cultured with 10 sclerotia without shaking. The controls of (ii) and (iii) were co-cultured without bacterial suspension. After 5 days of co-culture at 25°C, sclerotia were retrieved, dried by sterilized tissue, and cultured on WA plates for 5 days to evaluate the germination rate.


(2) \begin{eqnarray*}& \mathrm{Germination}\ \mathrm{inhibition}\ \left(\%\right)\notag\\&\quad =\left(1-\frac{\mathrm{Germination}\ \mathrm{rate}\ \mathrm{of}\ \mathrm{treatment}}{\mathrm{Germination}\ \mathrm{rate}\ \mathrm{of}\ \mathrm{control}}\ \right)\times 100\%,\notag\\&\kern0.5em \mathrm{where}\ \mathrm{Germination}\ \mathrm{rate}\ \left(\%\right)\notag\\& \quad=\left(\frac{\mathrm{Survived}\ \mathrm{sclerotia}}{10\ \mathrm{sclerotia}}\ \right)\times 100\% \end{eqnarray*}

(3) \begin{eqnarray*} &\mathrm{{Sclerotia}}\hbox{-}\mathrm{{derived}}\ \mathrm{mycelia}\ \mathrm{inhibition}\ \left(\%\right)\notag\\&\quad =\left(1-\frac{\mathrm{Colony}\ \mathrm{diameter}\ \mathrm{of}\ \mathrm{treatment}}{\mathrm{Mean}\ \mathrm{diameter}\ \mathrm{of}\ \mathrm{control}}\ \right)\times 100\% \end{eqnarray*}

As for (iv), 100 μl of bacterial suspension after being co-cultured for 5 days was serially diluted on plates for quantifying CFU. The controls consisted of 10 sterile glass beads instead of sclerotia. All these four assays were independently repeated three times, and each time had three biological replicates.

### Whole genome sequencing and analyses for *Trinickia sclerotiorum* sp. nov. MC

Total genomic DNA of TsMC was extracted by Presto gDNA Bacteria Advanced Kit (Geneaid), followed by size selection with the Short Read Eliminator XS kit (PacBio) to remove DNA fragments smaller than 10 kb. DNA concentration was measured using a Qubit 4.0 fluorometer (Thermo Fisher Scientific), and fragment size distribution was assessed with the Qsep 100™ system (BiOptic Inc., New Taipei City, Taiwan). Long-read sequencing libraries were constructed using a transposase-based method using the Rapid Barcoding Kit 96 V14 (SQK-RBK114.96) (Oxford Nanopore Technologies, Oxford, United Kingdom), followed by adapter attachment with a rapid adapter, in accordance with the manufacturer’s instructions. DNA libraries were then purified using short fragment buffer washes to enrich fragments larger than 1 kb. Sequencing was performed on a FLO-PRO114M flow cell (R10.4.1) using the ONT PromethION 24 platform. The raw sequencing data were processed using Dorado’s super-accurate basecalling model (400 bps, v7.6.8). The sequencing results were verified using NanoPlot [41] to validate the read length profile. Next, the raw reads were assembled into primary contigs using Flye [42]. The quality of the fully polished contigs was assessed using QUAST [43], whereas the completeness of the genome was evaluated using BUSCO [44]. Prokka [45] with default settings was used to predict open reading frames and locate tRNA and rRNA regions. Additionally, gene annotation was performed by aligning the sequences against the Refseq, eggNOG, KOfam, CARD [46–48] databases using DIAMOND [49] and HMMER [50]. Secondary metabolite biosynthesis-related gene clusters (BGCs) were predicted by the antiSMASH 7.0 [51]. Maximum likelihood phylogenetic trees were built for TsMC, together with other *Trinickia* species, using concatenated 16S rRNA-*gyrB*-*recA* gene sequences, and 120 621 polymorphic sites from 302 core genes identified with Panaroo v1.3.4 [52] and recombination-filtered with Gubbins v3.4.3 [53]. Sequences were aligned with MAFFT and constructed with IQ-TREE, assessed with 1000 bootstrap replicates. For sequence identity, average nucleotide identity (ANI) and digital DNA–DNA hybridization (dDDH) values between TsMC and other *Trinickia* species were calculated using FastANI v1.34 [54] and the GGDC web server v3.0 [55], respectively.

### Statistics

All statistical analyses were performed in the R version 4.5.1. Data normality was assessed using the Shapiro–Wilk test and Q–Q plots, and homogeneity of variance was assessed using the Levene’s test. Parametric data were analyzed using the *t-*tests or ANOVA, followed by the Tukey’s HSD *post hoc* test. For data violating these assumptions, the Yeo–Johnson transformation (or arcsine transformation for percentage data) was applied. For other data that still failed to satisfy normality or homoscedasticity, nonparametric Wilcoxon rank-sum test or Kruskal–Wallis test followed by the Dunn’s *post hoc* test were performed. For the mortality rates of sclerotia, which contained a high frequency of 0 and 1 values, the aligned rank transform procedure [56] was applied to factorial ANOVA at each timepoint, followed by the Tukey’s HSD *post hoc* test for each treatment, and the effect size of each factor was calculated as the proportion of variance explained, utilizing the *F* statistic–based weighting approach [57].

## Results

### Microbial effects contribute to sclerotia mortality

**Figure 1:**
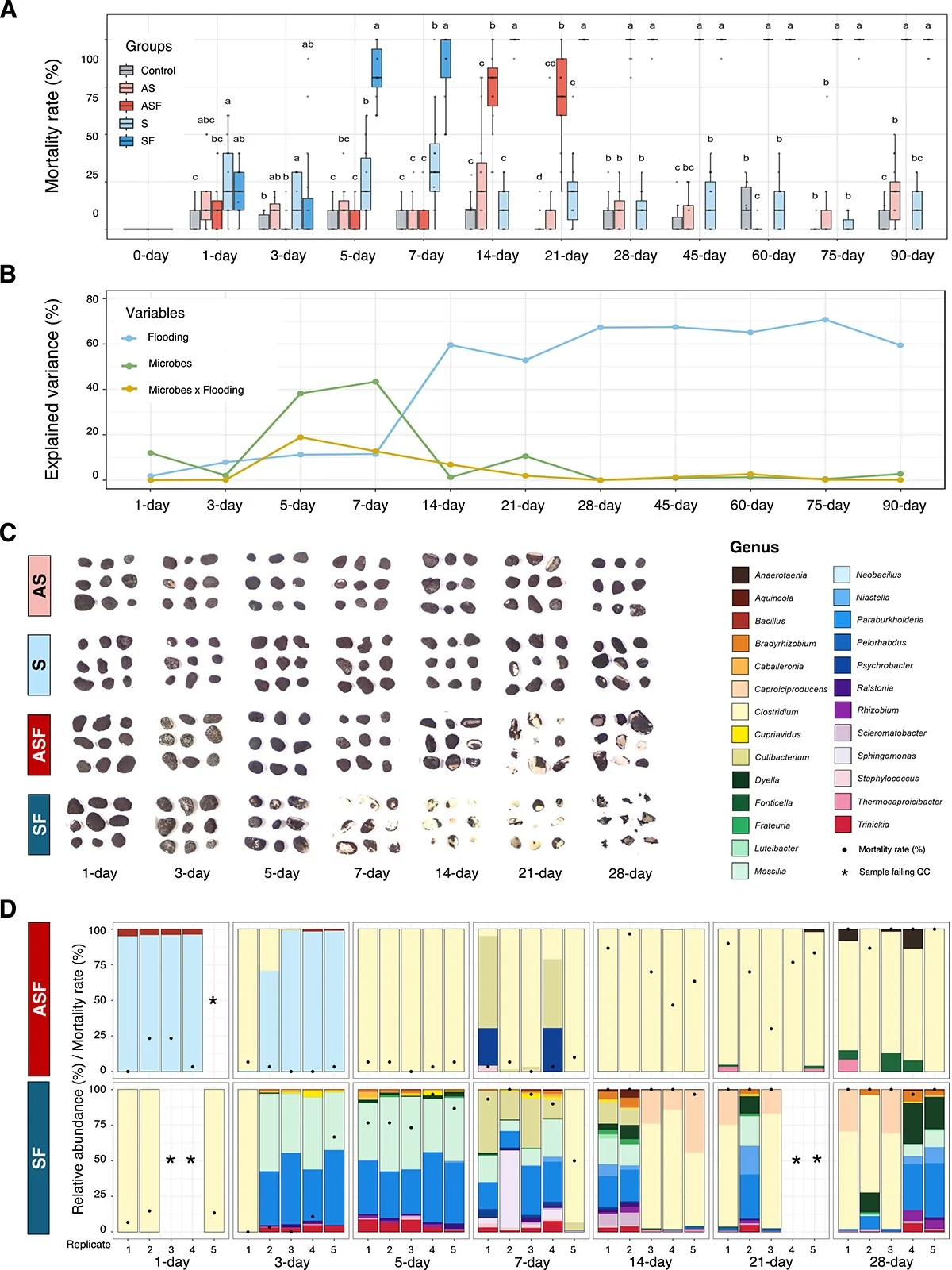
Microbial and flooding effects on sclerotia mortality. (A) Sclerotia mortality over 3 months in the treatment of control, autoclaved soil (AS), soil (S), autoclaved soil with flooding (ASF), and soil with flooding (SF). (B) Explained variance of the microbial, flooding, and interaction effects on sclerotia mortality. The microbial effect became apparent when sclerotia were buried for 5-day and 7-day. (C) Sclerotia morphology under different treatments. Sclerotia under flooding conditions (both ASF and SF) began to slough, and in the presence of microbes (SF), the slough of sclerotia can be observed when sclerotia were buried for 7-day. (D) PacBio full-length 16S rRNA sequencing for profiling the microbial composition of sclerotia. Autoclaving drastically reduced microbial diversity of sclerotia, and anaerobic bacteria became predominant over time. In contrast, SF exhibited diverse microbial composition that was potentially associated with sclerotia mortality when sclerotia were buried for 5-day and 7-day. Dot indicates the mean of sclerotia mortality for each biological replicate, and asterisk indicates low quality samples that were not passed for sequencing.

To understand the microbial effect on sclerotia mortality, a 3-month burial experiment in the autoclaved soil (AS) or conventional soil (S) with flooding or not (ASF or SF, respectively) was performed. Sclerotia mortality remained lower than 10.0% for the nonflooding groups (AS and S) (Fig. 1A). In contrast, under flooding condition, sclerotia mortality of the ASF and SF groups gradually increased to 100.0% when sclerotia were buried for 5-day to 28-day, with visible slough of the melanized rind and shattering of sclerotia (Fig. 1C). Specifically, sclerotia mortality of the SF group exhibited an earlier increase, starting from burial time 5- to 7-day; whereas sclerotia mortality of the ASF group increased when sclerotia were buried for 14-day to 21-day. This temporal delay due to the autoclaving treatment suggests that soil microbes play roles in enhancing the sclerotia mortality under flooding condition, particularly for soil bacteria that colonize sclerotia during the burial time between 5-day and 7-day (Fig. 1B; Table S1). Collectively, these findings suggest that the flooding treatment alone substantially contributes to sclerotia mortality, and the presence of soil microbes would synergistically accelerate sclerotia mortality.

**Figure 2:**
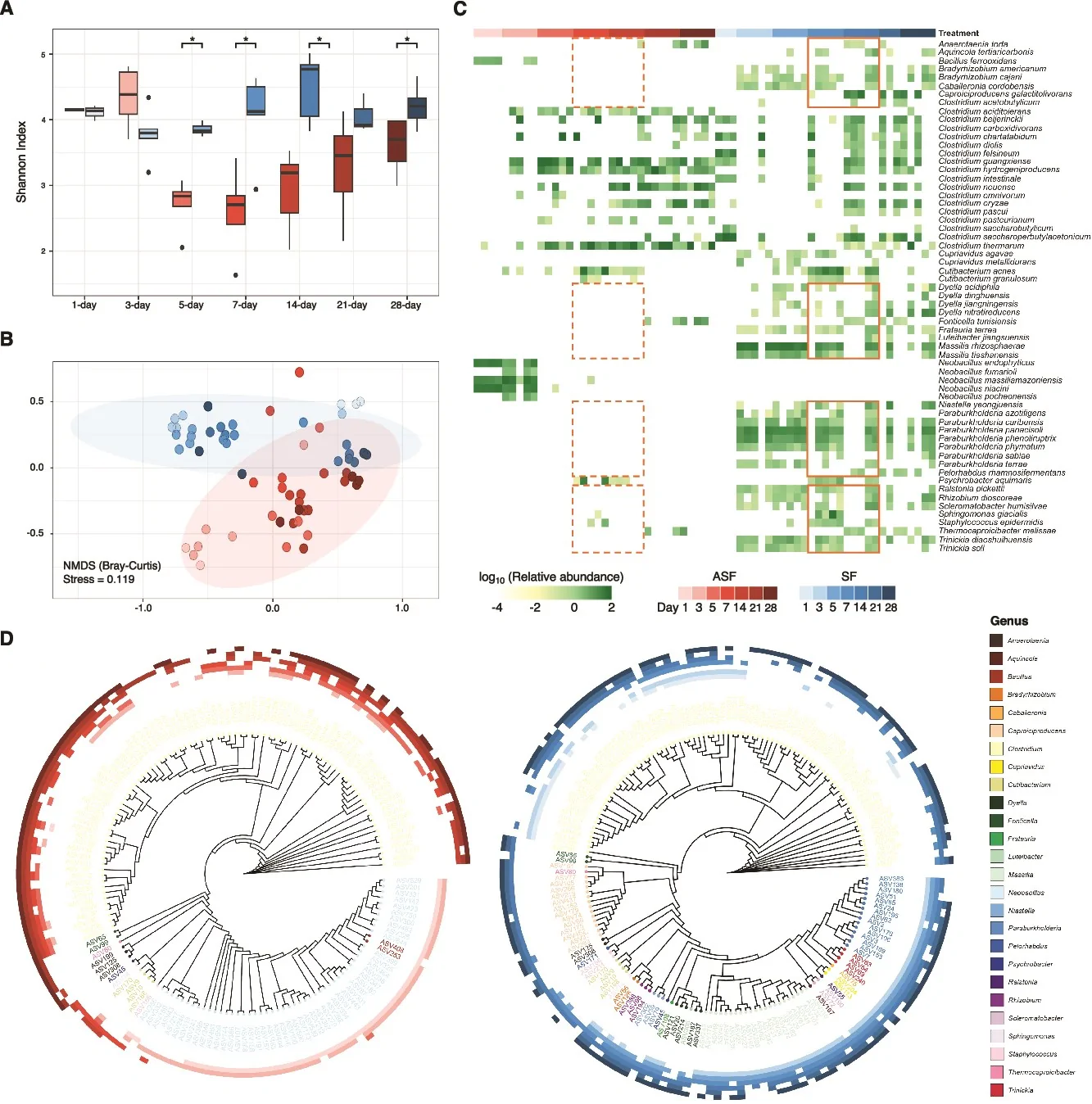
Diversity and abundance analyses of sclerotia microbiome. (A) α-Diversity according to the Shannon Index. (B) β-Diversity based on NMDS using the Bray–Curtis dissimilarity. (C) Microbial abundance showing by heatmap in the treatment of autoclaved soil with flooding (ASF) and soil with flooding (SF). The orange blocks highlight the microbes that are visually deficient in the ASF (dashed lines) and abundant in the SF (solid lines) when sclerotia were buried for 5-day and 7-day. (D) Maximum likelihood phylogeny of all sclerotia-associated bacteria of the ASF and SF treatments.

To identify the sclerotia-associated bacteria that contribute to sclerotia mortality, we applied PacBio full-length 16S rRNA sequencing and bulk microbiome analysis to elucidate the temporal dynamics of the sclerotia microbiome. A total of 4029 ASVs were obtained from 65 samples, with 3574 ASVs matched at the genus level (≥94.5% identity) and 1575 ASVs assigned at the species level (≥98.7% identity) according to the NCBI 16S RefSeq database. In the analyses of α-diversity, the Shannon Index for burial time from 5-day to 14-day were significantly lower in the ASF group than in the SF group (Fig. 2A; Table S2). In terms of β-diversity, the NMDS and PERMANOVA revealed two discernible clusters for the ASF and SF groups, as well as distinct temporal trajectories within each group (Fig. 2B; Table S3). The total variance explained by the microbial effect, incubation time, and their interaction was 9.9%, 18.0%, and 13.9%, respectively. Collectively, these results indicate that both microbial effect and burial time could influence the structure of the sclerotia microbiome.

### Microbial diversity and differential abundance analyses of sclerotia microbiome

In addition to the temporal dynamics, the taxonomic composition of the sclerotia microbiome was also different between the ASF and SF groups. Focusing on core ASVs (average abundance >0.5% & occupancy >10%), there were 252 ASVs assigned to 26 genera and 59 species (Fig. 2C). The ASF group (151 ASVs) harbored 10 genera and 28 species, and the SF group (174 ASVs) harbored 24 genera and 53 species (Fig. 2D). For the overall bacterial diversity, *Bacillota* was dominant at 96.0% in the ASF group, compared to 60.3% in the SF group. Conversely, *Pseudomonadota* was higher in the SF group at 35.6% than in the ASF group at 1.3%. Minor differences were observed in *Actinomycetota* (2.6% in ASF vs. 2.3% in SF) and *Bacteroidota* (0.0% in ASF vs. 1.7% in SF). At the class level, the ASF group was dominated by *Clostridia* (60.3%) and *Bacilli* (35.8%). As for the SF group, *Clostridia* (58.6%) was also the most prevalent class, followed by *Betaproteobacteria* (27.6%), *Gammaproteobacteria* (4.6%), and *Alphaproteobacteria* (3.4%).

**Figure 3:**
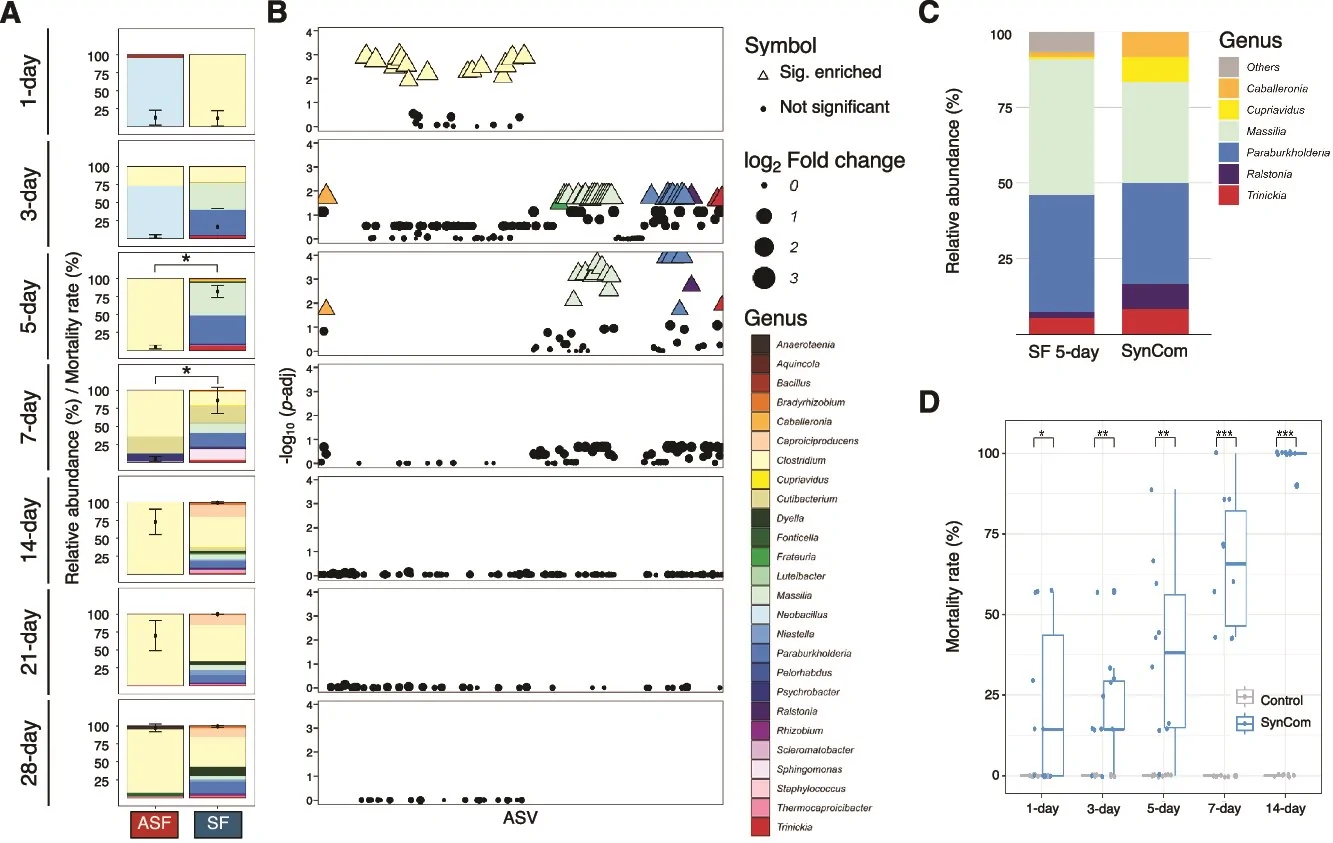
Differential abundance analysis and SynCom assembly. (A) Sclerotia mortality in the treatment of autoclaved soil with flooding (ASF) and soil with flooding (SF) over time. Values indicate the averaged mortality ± standard deviation. Asterisks indicate significant difference in mortality when sclerotia were buried for 5-day and 7-day. The bar panels of ASF and SF were colored by the proportion of microbial diversity. (B) Differential abundance analysis based on ANCOM-BC2. There are significantly enriched ASVs when sclerotia were buried for 3-day and 5-day but not 7-day. Because the sclerotia mortality of 3-day was not yet significantly different between ASF and SF, the increase of ASVs when sclerotia were buried for 3-day and 5-day may contribute to the mortality of 5-day and 7-day. Collectively, the 5-day SF sclerotia microbiome was the target of mimicry. (C) A SynCom of 12 bacteria was assembled to mimic not only the microbial composition (taxonomically) but also the antagonistic potentials (functionally). The SynCom includes one *Caballeronia* sp*.*, one *Cupriavidus* sp., four *Massilia* spp*.*, four *Paraburkholderia* spp., one *Ralstonia* sp., and one *Trinickia* sp. (D) The SynCom-treated sclerotia exhibited higher mortality rate over time, and none survived after being co-cultured for 14 days.

Relative abundance analysis showed that the autoclaving treatment eliminated the majority of bacteria and enriched the thermotolerant *Bacillota* from sclerotia in the ASF group (Fig. 1D). Specifically, *Neobacillus* dominated for burial time 1-day (96.5%) but was gradually superseded by *Clostridium* starting from 3-day, indicating a shift toward anoxic conditions. In contrast, the sclerotia microbiome of the SF group displayed greater diversity and more sophisticated community shifts. Although *Clostridium* comprised 100.0% of the community for burial time 1-day, three genera (*Massilia, Paraburkholderia*, and *Trinickia*) within *Pseudomonadota* became dominant with prolonged burial time (Fig. 3A). The microbial dynamics in the SF group for burial time 5-day to 7-day aligned with the acceleration of sclerotia mortality, and the enriched ASVs based on the ANCOM-BC2 identified 17 ASVs belonging to six genera and 12 species (Fig. 3B), including *Caballeronia, Frateuria, Massilia, Paraburkholderia, Ralstonia*, and *Trinickia*. Overall, the results uncovered the temporal dynamics of the sclerotia microbiome and pointed out that the enriched bacteria genera coincided with the rapid increase of sclerotia mortality.

### Twelve bacteria were isolated from sclerotia for SynCom assembly

To investigate causality between the enriched bacteria and sclerotia mortality, we assembled a SynCom to mimic the 5-day SF microbiome. A total of 165 morphologically distinct bacterial strains were isolated from sclerotia in the SF group at the 5-day burial timepoint. Based on Sanger sequencing of full-length 16S rRNA gene sequences, 52 strains matched the microbiome ASV dataset at the genus level (≧94.5% identity), and 40 strains matched at the species level (≧98.7% identity). Among these strains, 12 strains matching nine enriched ASVs (≧97.0% identity) based on the ANCOM-BC2 and DESeq2 at 5-day were selected for SynCom assembly, including *Caballeronia zhejiangensis* N8 (ASV75), *Cupriavidus* sp. N4 (ASV113), *Massilia* sp. R20 (ASV249), *Massilia* sp. TB221 (ASV49), *Massilia* sp. RB201 (ASV249), *Massilia* sp. RB202 (ASV249), *Paraburkholderia* sp. I59 (ASV2), *Paraburkholderia phenoliruptrix* TB211 (ASV2), *Paraburkholderia phymatum* R7 (ASV51), *Paraburkholderia* sp. T17 (ASV178), *Ralstonia* sp. R4 (ASV55), and *Trinickia* sp. MC (ASV69) (Fig. S2; Table S4). At the genus level, the SynCom comprises six genera accounting for 93.4% of total community abundance (Fig. 3C); at the species level, the SynCom comprises 11 species (≥98.7% 16S rRNA identity to a described species) accounting for 56.3% of total abundance (Table S5), comparable to coverage reported in previous SynCom studies [58, 59]. When sclerotia were inoculated with the SynCom, sclerotia mortality was significantly increased after 1-day co-culture with the SynCom, gradually reaching 100.0% mortality by 14-day co-culture, whereas the control remained fully viable throughout (Fig. 3D).

### Spatial microbiome uncovers the invasion pattern of SynCom

**Figure 4:**
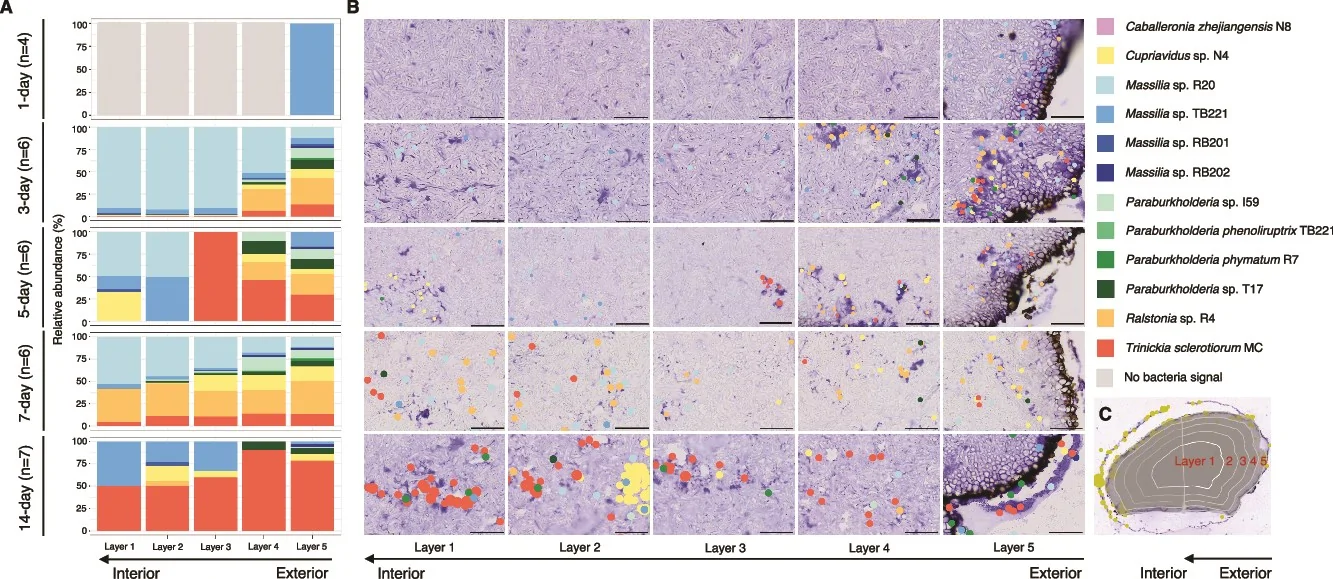
Spatiotemporal distribution of SynCom members within sclerotia. (A) The averaged relative abundance of SynCom members in different layers of sclerotia. Few bacteria were detected in the interior layer 1–4, suggesting the bacterial invasion was not yet progressive when sclerotia were co-cultured for only 1-day. Subsequently, *Massilia* spp. were the leading species that reach to the core of sclerotia although the sclerotia mortality remained low when sclerotia were co-cultured for 3-day. *Cupriavidus* sp. N4, *Ralstonia* sp. R4, and *Trinickia sclerotiorum* MC (TsMC) spread toward the interior layers when sclerotia were co-cultured for 5-day and 7-day, which was the stage that sclerotia mortality began to increase. (B) After sclerotia were co-cultured for 14-day, no sclerotia remained viable and the abundance of TsMC increased drastically over other SynCom members, indicating its importance to sclerotia mortality. (C) Schematic diagram of five concentric zones with equal area on a sclerotium, ranging from the interior core (layer 1) to exterior periphery (layer 5).

To visualize and quantify the spatiotemporal association between bacterial invasion and sclerotia mortality (Fig. 4A), we applied the 10× Xenium *in situ* spatial platform, together with crystal violet staining to SynCom-treated sclerotia (Fig. 4B). After 1-day co-culture, bacteria were detected only on the most exterior sclerotia layer (layer 5), with *Massilia* sp. TB221 as the predominant SynCom member. By 3-day co-culture, *Massilia* sp. TB221 and *Massilia* sp. R20 had reached the interior sclerotia (layer 1–4) (Fig. 4C), and small numbers of *Cupriavidus* sp. N4, *Ralstonia* sp. R4, and *Trinickia* sp. MC were also detected in exterior sclerotia (layer 4–5) at low relative abundance. At 5- to 7-day co-culture, the relative abundance of *Ralstonia* sp. R4 and *Trinickia* sp. MC increased progressively in the interior layers, coinciding with the stage at which mortality of SynCom-treated sclerotia increased sharply (Fig. 3D). Although the relative abundance of most *Paraburkholderia* spp. increased inside sclerotia, they did not reach layer 1 and 2. By 14-day co-culture, when sclerotia viability was completely suppressed, the relative abundance of *Trinickia* sp. MC exceeded that of all other SynCom members simultaneously across layer 1–5. Collectively, Xenium spatial analysis uncovered the spatiotemporal invasion pattern of the SynCom, revealing the distinct spatial distribution and antagonistic potential of each SynCom member.

### Individual antagonistic assays and SynCom drop-out test confirmed that *Trinickia* sp. MC is the key bacteria for sclerotia mortality

**Figure 5:**
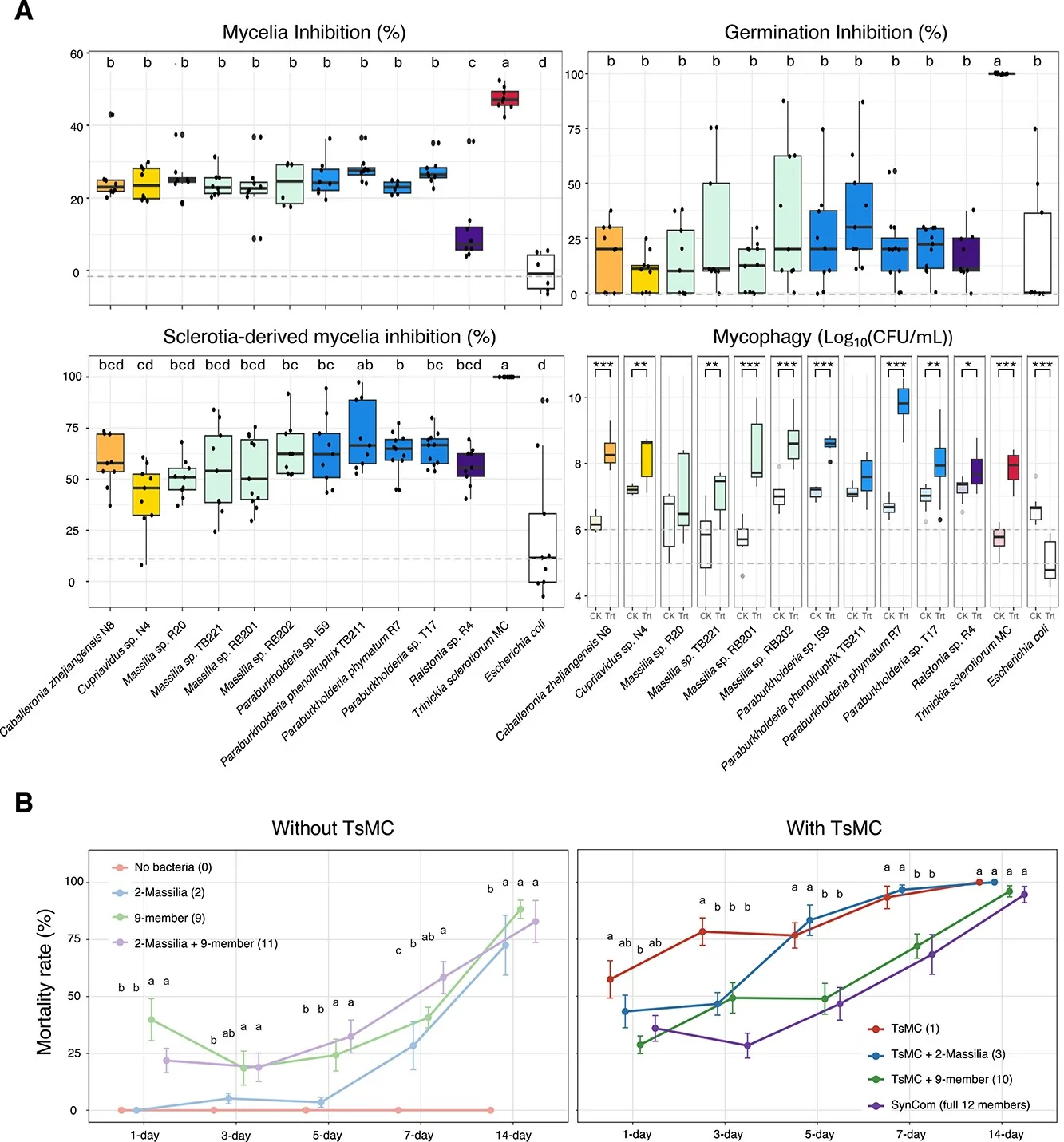
Four antagonistic assays and SynCom drop-out test. (A) Antagonistic abilities of the 12 SynCom members and a reference bacterium, *Escherichia coli*, were evaluated across four assays. Mycelia inhibition measured on 1/10 TSA was shown. (B) Sclerotia were treated with different combinations of SynCom members based on the presence or absence of three groups: *Trinickia sclerotiorum* MC (TsMC) alone, *Massilia* sp. R20 together with *Massilia* sp. TB221 (2-Massilia), and the remaining nine SynCom members (9-member). Treatments with (right) and without (left) TsMC are shown in separate line charts. The number in brackets of each label indicates the number of bacterial species included in the treatment.

To evaluate the individual potential of these 12 SynCom members for fungal antagonism, we further conducted four different antagonistic assays (Table S6). In the mycelia inhibition assay, only *Trinickia* sp. MC produced clear inhibition zones, regardless of the culture media used. In the germination inhibition and sclerotia-derived mycelia growth assays, *Trinickia* sp. MC demonstrated complete inhibition, exhibiting robust antagonistic power. The remaining 11 bacteria showed no significant germination inhibition, even though some of them could suppress the sclerotia-derived mycelia growth. Lastly, 10 bacteria (except for *Massilia* sp. R20 and *P. phenoliruptrix* TB211) increased their population in the mycophagy assay, suggesting that most strains were capable of utilizing sclerotia as a nutrient source (Fig. 5A).

Because *Massilia* sp. R20 and *Massilia* sp. TB221 (2-Massilia) were the earliest colonizers inside sclerotia, and *Trinickia* sp. MC (TsMC) has the strongest antagonistic capability and it was the colonizer closely coincided with sclerotia mortality according to the spatiotemporal microbiome (Fig. 4A), 2-Massilia and TsMC were dropped from the full SynCom to validate their contributions to sclerotia mortality (Fig. 5B). The result showed that TsMC was the most dominant contributor to sclerotia mortality (Table S7) because dropping TsMC significantly decreased mortality and its mono-inoculation alone could accelerate sclerotia mortality (Fig. 5B). In contrast, 2-Massilia caused no difference at early stages and a lower mortality after 14 days. Also, the combination of 2-Massilia and TsMC did not show synergism or attenuation to the effect of TsMC. While the remaining bacteria of the full SynCom (9-member) exhibited weaker antagonism along the timeline, the 9-member could attenuate the antagonistic effect of TsMC when treated together. These observations suggested that bacteria–bacteria interactions in the SynCom together contributed to overall sclerotia mortality and TsMC is the apparent key species.

### Genome analyses and species identification for *Trinickia sclerotiorum* sp. nov

To reveal the species identity and explore the antifungal properties of TsMC, we completed the whole genome sequencing via the platform of Oxford Nanopore Technologies (Fig. S3). Phylogenetic analysis based on 302 core genes placed TsMC in a clade distinct from all 13 known *Trinickia* species. In addition, the comparison of ANI values between TsMC and other *Trinickia* species ranged from 80.7% to 81.8%, and the comparison of dDDH values ranged from 22.3% to 23.4% (Table S8). These results indicate TsMC does not belong to any known *Trinickia* species and thus we describe this strain as a new species, *Trinickia sclerotiorum* sp. nov. type strain MC. Genome mining of TsMC discovered biosynthetic gene clusters associated with antimicrobial properties such as ornibactin and rhizomides (Table S9), pointing to possible antagonistic mechanisms and its potential as a novel biocontrol agent.

## Discussion

Sclerotia are produced by a broad range of fungi, including at least 45 genera in the *Ascomycota* and 40 genera in the *Basidiomycota* [60]. These dormant fungal structures are rich in polysaccharides, and their compact architecture confers exceptional tolerance to environmental stresses, enabling long-term nutrient storage and survival. Among these, the ascomycete *S. sclerotiorum* is a major sclerotia-forming plant pathogen responsible for annual agricultural losses of up to $200 million in the USA and $600 million in the Canadian canola industry [61, 62]. The sclerotia of *S. sclerotiorum* are encased in a hard, melanized rind, rendering them highly resistant to ultraviolet radiation, adverse environmental conditions, and chemical treatments [63]. Under favorable conditions, sclerotia can germinate either myceliogenically or carpogenically, enabling direct host infection or long-distance dispersal to new fields [64]. Previous studies have identified flooding as a critical factor influencing sclerotia survival [25, 65, 66]; however, the role of microbial activity under flooding conditions has received comparatively little attention. Some studies have proposed that reduced sclerotia survival under warm and wet conditions resulted from enhanced microbial decomposition [67]. Similarly, it has been reported that water exposure caused nutrient leakage from sclerotia, thereby facilitating microbial colonization [68]. Other studies also demonstrated that soil microbes can produce antifungal compounds that suppress sclerotia survival specifically under anoxic conditions [25, 69]. In this study, we evaluated the flooding and microbial effects, and the results showed that sclerotia mortality was significantly accelerated in the presence of soil bacteria.

Our results showed that the sclerotia microbiome was dominated by *Pseudomonadota*, particularly members of the *Oxalobacteraceae* and *Burkholderiaceae* families, with their enrichment coinciding with sclerotia mortality. *Burkholderiaceae* are well known for their antifungal activities [70]. Members of *Burkholderia sensu lato*, including *Caballeronia, Paraburkholderia*, and *Trinickia*, are frequently isolated from fungal tissues [71], and several *Burkholderiaceae* taxa have been reported to proliferate in soils amended with sclerotia of *S. sclerotiorum* [72]. Likewise, *Oxalobacteraceae* members such as *Massilia* are consistently enriched in the hyphosphere and are recognized as keystone taxa in the fungal-associated microbial communities [73]. Beyond inhibiting mycelial growth, bacteria from both families have been shown to exhibit mycophagous behavior, enabling them to directly exploit fungal biomass [74, 75]. Collectively, the enrichment of *Burkholderiaceae* and *Oxalobacteraceae* within the sclerotia microbiome supports the likelihood of these bacteria in driving sclerotia mortality. Nevertheless, bulk microbiome analyses alone cannot resolve whether this effect arises from the collective activity of the microbial community or from a few key species.

The 10× Genomics Xenium *in situ* platform has been extensively validated for spatial gene expression analysis in human [76], mouse [77], and plant tissues [78]; however, its application to fungal tissue or bacterial detection has not previously been demonstrated. Here, we applied Xenium-based spatial detection of a bacterial SynCom within fungal sclerotia as a proof-of-concept. We described a newly identified species, *Trinickia sclerotiorum* sp. nov. type strain MC (TsMC), which colonized the most interior sclerotia, concomitant with the highest sclerotia mortality rate (Fig. 4). TsMC was further identified as the primary member driving sclerotia mortality via the SynCom drop-out test, and it also has the strongest antagonistic capability across all four antagonistic assays (Fig. 5, Table S6). Genome mining of TsMC revealed biosynthetic gene clusters associated with antifungal properties, consistent with previous reports suggesting that *Trinickia* species can produce antifungal compounds [79] and display mycophagous behavior toward chlamydospores of the mycorrhiza-like fungus *Serendipita indica* [80]. In contrast, although two *Massilia* members (*Massilia* sp. R20 and *Massilia* sp. TB221) were detected in the sclerotia core earlier than TsMC, their contribution to sclerotia mortality appeared limited. Moreover, although the remaining SynCom 9-member could increase sclerotia mortality over time, their relative abundance and spatiotemporal distribution were more limited inside sclerotia. The treatment of 9-member together with TsMC even attenuated the antagonistic effect of TsMC. Nonetheless, the 9-member and 11-member SynCom could eventually reach the same level of sclerotia mortality with extended time. Accordingly, under a field condition, biocontrol application with a single strong antagonistic species may benefit from a fast efficacy in a short window of exposure, and a complex community of multiple weak antagonistic species may still reach the same effect if the time of exposure is allowed. As our SynCom represents only 56.3% species-level bacterial abundance of 5-day SF microbiome, higher level of the complexity in the bacteria–bacteria interaction may not be captured in this study and should be aware for consideration.

In summary, our findings confirmed the participation of soil bacteria and described a novel species, *Trinickia sclerotiorum* sp. nov. type strain MC, for sclerotia mortality. By integrating the advantages of bulk microbiome using PacBio full-length 16S rRNA sequencing, SynCom, and 10× Genomics Xenium, the comprehensive framework presented in this study broadens current understanding of microbial ecology in a spatiotemporal perspective, providing a vital stepping stone toward studying bacteria-fungi interactions and exploring novel biocontrol agents.

## Supplementary Material

Supplementary_material_wrag201

## Data Availability

All sequence data, including the PacBio sequencing data of the sclerotia microbiome, and the whole-genome sequence of *Trinickia sclerotiorum* sp. nov. MC, are available in the NCBI database under BioProject PRJNA1475221 and Harvard Dataverse https://doi.org/10.7910/DVN/DEERTV.
