# Supplementary material for: Spatial microbiome and synthetic community analyses reveal Trinickia sclerotiorum sp. nov. promotes sclerotia mortality of Sclerotinia sclerotiorum
Source: ISME J. 2026 Jul 29;20(1):wrag201. doi: 10.1093/ismejo/wrag201 (PMC13615538; doi:10.1093/ismejo/wrag201)
Supplement: Supplementary file 1 [file supplementary_material_wrag201.zip › Supplementary_Figures_and_legends_wrag201.docx]

**Figure S1. Schematic workflows.** (A) Experimental setup and sampling timepoints over 3-month burial for sclerotia buried in control (CK), autoclaved soil (AS), autoclaved soil with flooding (ASF), soil (S), and soil with flooding (SF). (B) Half of sclerotia collected from 11 different burial times were used to evaluate the mortality rate, and the hyphae germinated from sclerotia were PCR-confirmed using the *Sclerotinia sclerotiorum*-specific primers. (C) Another half of sclerotia were extracted for DNA and sent for PacBio full-length 16S rRNA sequencing. The microbiome analyses were conducted by the DADA2 pipeline and bacteria species were identified by SILVA and NCBI databases. (D) The sclerotia microbiome of SF 5-day was mimicked to assemble a synthetic community (SynCom) with 12 bacteria members. The SynCom was inoculated to sclerotia to assess the microbial effect on mortality rate. The SynCom-treated sclerotia were also subjected to 10x Genomics Xenium *in situ* platform to quantify the spatial distribution of SynCom members.

**Figure S2. Maximum−likelihood phylogenetic placement of the 12 SynCom members in six bacterial genera.** (A) *Caballeronia*, (B) *Cupriavidus*, (C) *Massilia*, (D) *Paraburkholderia*, (E) *Ralstonia* and (F) *Trinickia*. Panels A, B, D, E and F are based on concatenated 16S rRNA gene−*gyrB*−*recA* sequences, while panel C (*Massilia*) is based on concatenated 16S rRNA gene−*gyrB*−*lepA* sequences. Numbers at nodes are Bootstrap values (%) after 1000 replicates. Isolates obtained in this study are shown in bold. The scale bar represents 0.05 substitutions per nucleotide site.

**Figure S3. Maximum likelihood phylogenetic trees of *Trinickia sclerotiorum* sp. nov. type strain MC and closely related *Trinickia* spp.** (A) The tree based on 120,621 polymorphic sites from 302 core genes. Numbers at nodes are Bootstrap values (%) after 1000 replicates. *Burkholderia cepacian* BC16 is included as an outgroup. The scale bar represents 0.08 substitutions per nucleotide site. (B) Whole genome circular map of *Trinickia sclerotiorum* sp. nov. type strain MC.
